# Girls in the boat: Sex differences in rowing performance and participation

**DOI:** 10.1371/journal.pone.0191504

**Published:** 2018-01-19

**Authors:** Kevin G. Keenan, Jonathon W. Senefeld, Sandra K. Hunter

**Affiliations:** 1 Department of Kinesiology University of Wisconsin–Milwaukee, WI, United States of America; 2 Center for Aging and Translational Research University of Wisconsin–Milwaukee, WI, United States of America; 3 Department of Physical Therapy Marquette University, Milwaukee, WI, United States of America; Norwegian University of Science and Technology, NORWAY

## Abstract

Men outperform women in many athletic endeavors due to physiological and anatomical differences (e.g. larger and faster muscle); however, the observed sex differences in elite athletic performance are typically larger than expected, and may reflect sex-related differences in opportunity or incentives. As collegiate rowing in the United States has been largely incentivized for women over the last 20 years, but not men, the purpose of this study was to examine sex differences in elite rowing performance over that timeframe. Finishing times from grand finale races for collegiate championship on-water performances (n = 480) and junior indoor performances (n = 1,280) were compared between men and women across 20 years (1997–2016), weight classes (heavy vs. lightweight) and finishing place. Participation of the numbers of men and women rowers were also quantified across years. Men were faster than women across all finishing places, weight classes and years of competition and performance declined across finishing place for both men and women (*P*<0.001). Interestingly, the reduction in performance time across finishing place was greater (*P*<0.001) for collegiate men compared to women in the heavyweight division. This result is opposite to other sports (e.g. running and swimming), and to lightweight rowing in this study, which provides women fewer incentives than in heavyweight rowing. Correspondingly, participation in collegiate rowing has increased by ~113 women per year (*P*<0.001), with no change (*P* = 0.899) for collegiate men. These results indicate that increased participation and incentives within collegiate rowing for women vs. men contribute to sex differences in athletic performance.

## Introduction

Men are generally faster than women for reasons that consist of anatomical and physiological sex differences including: 1) larger and more powerful muscle mass [1–3], 2) higher maximal oxygen consumption [4,5], and 3) increased biomechanical efficiency [6,7]. However, the differences between men and women in performance are greater than what would be expected due to anatomical and physiological differences alone. For example, sex differences in elite sport performance widen with finishing place in almost every sport previously studied, including: 1) short-, long-, and ultra-distance running [8–12], 2) triathlon [13,14], 3) speed-skating [15], 4) cycling [14], and 5) in most swimming events [8,14].

Two prevailing hypotheses have been suggested for explaining the greater sex difference in performance (than due to physiology and anatomy) with increased finishing place, the *sociocultural conditions hypothesis* and the *evolved predispositions hypothesis* [16–18]. The *sociocultural conditions hypothesis* suggests that decreased opportunities and participation are two major contributors to the lack of depth among women in sports and explains the larger than expected sex difference in performance [9,10]. Historically, women have had less opportunity to participate in sport within the United States, especially prior to the passage of Title IX in 1972. Title IX is a federal law that legislates equal opportunity for women in educational settings, including athletics. The *sociocultural conditions hypothesis* is supported by significant relationships existing between decreased participation rates in sport and increased sex differences [8,9,12]. Swimming is a sport where participation rates have been more similar historically between men and women than other sports, for example, distance running [8,19]. Consequently, the sex difference in swimming is less than marathon running [8], again supporting the *sociocultural conditions hypothesis* and the role of increasing participation to minimize sex differences.

Conversely, the *evolved predispositions hypothesis* suggests that sex differences occur not due to sociocultural conditions, but because there exists an evolutionary difference between the sexes that drives a difference in competitiveness [16–18,20]. This hypothesis holds that, although sociocultural conditions modulate the expression of sex differentiated behavior, sex differences in competitiveness would occur even if society were egalitarian [16]. Evidence provided in support of this hypothesis includes the lack of an absolute or relative increase in the number of “fast” female distance runners since the mid-80s [16], large sex differences in sport participation even since Title IX [21], and an actual widening of the sex difference in elite short duration events [15]. Importantly, the *evolved predispositions hypothesis* leads to very specific, testable and expected results, specifically: 1) that sex differences should not change substantially across time or finishing place even given large changes in participation; and 2) that the sex difference in participation and performance will be larger for team than individual sports. This second prediction suggests that team sports require both motivation to engage in physical competition and motivation to engage in cooperative group challenges, with both factors reported to be greater in males than females [21–23].

Rowing is unique among team sports and given the sex specific changes in the sport over the last 20 years, it provides a testbed to evaluate predictions that arise from the *sociocultural conditions* and *evolved predispositions hypotheses*. Rowing is the only team sport evaluated by performance time sanctioned by the National Collegiate Athletic Association (NCAA) for women but not men. The premier event in rowing is the eight boat with a coxswain (8+), which involves 8 rowers working together as a team under the guidance of one coxswain to traverse a 2000 m distance as fast as possible. Indeed, the determination of a collegiate national champion in men’s and women’s rowing is weighted most heavily towards the performance of the 8+, with individual performances in single sculls not even used in calculating a collegiate team champion. Furthermore, collegiate women’s rowing programs have experienced arguably larger relative gains in participation than any other sport since Title IX was enacted in 1972. For example, since 1997 when women’s rowing became an NCAA sport, the total number of collegiate women’s teams has increased from 98 to 146 teams [24]. In contrast, men’s rowing is not an NCAA-sanctioned sport and participation in collegiate sanctioned rowing has declined from 90 to 58 teams over the same time period [24]. Interestingly, no single female collegiate sport offers more athletic scholarships than women’s rowing (20 scholarships), while far fewer men’s rowing programs offer any athletic scholarships. Furthermore, NCAA regulations related to student athletes and the sport of rowing have not changed dramatically over the last 20 years, creating relatively homogenous cohort characteristics and similar exposure to training across the 20 years. For example, at least some sociodemographic characteristics are regulated by the NCAA and have remained similar for the past 20 years, including that student athletes must be enrolled in an accredited university, demonstrate academic progress (GPA >2.0/4.0), and conclude competing within 7-years of graduating high school. Additionally, collegiate rules tightly regulate the training hours and use of performance enhancing drugs among student-athletes, thus, the exposure to training is likely similar between men and women and across the 20-year time period. We used rowing and its recent history of change in opportunities for men and women as a unique model to determine whether altered participation of men and women within a sport influences the sex difference in performance. The *primary* purpose of this study was to determine the change in collegiate rowing performance of men and women from 1997 to 2016 in heavy and lightweight classes. We assessed this by analyzing the finishing times of the top 6 boats (grand finale finishers) for men and women competing in their respective collegiate championships for Division 1 rowing from 1997–2016. Consistent with the *sociocultural conditions hypotheses*, we expected that women would improve in rowing performance at a greater rate than men over the 20-year timespan. Moreover, given the increased depth in participation for women over that 20-year timespan, we expected that the performance across place would not only narrow between men and women, but that women would outperform men (relatively) at higher finishing places, further supporting the *sociocultural conditions hypothesis*. Interestingly, the NCAA does *not* sponsor women's lightweight rowing championships and athletic scholarship opportunities for lightweights are fewer. Thus, we expected that the greater relative performance by women at higher finishing places would apply only to heavyweight rowing and not to lightweight rowing.

In addition, given the increased opportunities provided to women vs. men athletes in terms of scholarships to compete in collegiate rowing, our *secondary* purpose was to evaluate the corresponding sex differences in high school rowing from 1997 to 2016. Notably, elite rowing performance is difficult to assess at the high school level as many of the best performers may be on teams without other elite performers, which complicates analyzing team performances. Therefore, we analyzed individual performances at the high school level utilizing the metric most commonly used by colleges while recruiting high school rowers, specifically the time to complete a 2000 m distance on a rowing ergometer (i.e., “erg time”). We assessed ergometer times over the same 20-year timeframe (1997–2016) using the top 16 finishing times for junior (i.e., high school) men and women competing in the World Indoor Rowing Championships (C.R.A.S.H.-B Sprints; Boston, MA). Again, consistent with the *sociocultural conditions hypotheses*, we expected that the sex difference in rowing times would decrease over the intervening 20-year timespan and would widen with finishing place for men vs. women.

## Materials and methods

### Primary purpose

Finishing times of the top 6 boats for men and women competing in their respective Division 1 collegiate championships were obtained from an online database (http://www.row2k.com/) and 20 years of competition data (1997–2016) were collected. Heavyweight women (> 59 kg) compete at the NCAA Championships and both lightweight men and women, as well as heavyweight men (> 72.5 kg), compete in the Intercollegiate Rowing Association (IRA) Championships; collectively, these events will be referenced as ‘Collegiate Championships’. Data were collected from the top 6 boats of each race because each Grand Finale contained at least 6 boats. On rare occasion (3 races), 7 boats competed in the Grand Finale and the 7^th^ boat was removed from analyses due to the small sample size. Because the 8+ is the premier race in rowing and most largely contributes to overall team scoring, only the 8+ boat was included in analyses. Thus, 480 data points were collected for finishing time and subsequent calculations (two sexes × two weight classes × six finishing places × 20 years of competition).

### Secondary purpose

Finishing times of the top 16 men and women competing in the World Indoor Rowing Championships for the Junior division and two weight classes (lightweight and heavyweight) were obtained from an online database (http://www.crash-b.org/) and 20 years of competition data (1997–2016) were collected. Data were collected from the top 16 finishers of each race, because for several years only 16 individuals were included in the final round of competition. Thus, 1,280 data points were collected for finishing time (two sexes × two weight classes ×16 finishing places × 20 years of competition).

Rowing times were expressed in minutes (min) for each sex, weight class, place, and year. To determine the change in performance across finishing place, all times were made relative to 1^st^ place for that year of competition within the same race (i.e. same sex and weight class), as we have done previously [8,12].

Collegiate participation data was also collected from a publication from the NCAA [24] which included data for men and women combined for heavyweight and lightweight rowing. Thus, comparisons across the two weight classes was not possible. Junior participation data for men and women and heavyweight and lightweight rowing was collected from an online database in which the performance data during the World Indoor Rowing Championships was also collected (http://www.crash-b.org/).

Sex differences in performance were calculated by converting finishing times (min) into velocities (divide 2000 m by finishing time; m·min^-1^), and making women’s velocity relative to men’s: (men’s velocity–women’s velocity) / men’s velocity × 100%. Sex differences in performance were calculated for indoor rowing performance times because times were performed under similar conditions—i.e. similar ergometers, same date, same performance facility, etc. For the Collegiate Championships, sex differences in performance were not calculated because heavyweight women performed on a different date, course, city, and weather conditions at the NCAA Championships compared to the lightweight men and women and heavyweight men at the IRA Championships.

The data were thoroughly checked for errors in online data entry and performance outliers, and one case was identified as problematic (6^th^ place during 2000 IRA Championships for lightweight women). That performance was removed from all analyses as that team had a recorded performance time of 9.8 minutes in the grand finale while also having an average performance time in two preliminary rounds of 7.0 minutes, demonstrating either a failure of the boat or the athletes during the finals. Nonetheless, removal of that performance time did not impact significance for any statistical test.

### Statistical analysis

Data were reported as mean ± SD within the text and tables and displayed as mean ± SEM in figures. Statistical analysis was performed using the Statistical Package for the Social Sciences (version 24.0; SPSS Inc., Chicago, IL). Separate univariate analysis of variances (ANOVAs) were used to determine the sex difference in mean rowing time and sex difference in performance across years of competition (1997–2016) and finishing places (1^st^ to 6^th^ or 1^st^ to 16^th^ for on-water and indoor rowing, respectively). Post hoc analysis (pair-wise comparisons) were used to test for differences among pairs within a data set when significant main effects were identified. Separate univariate ANOVAs were used to determine changes in performance across the finishing places and year of competition. Post-hoc ANOVAs were performed with corrected *P*-values for (*P < 0*.*025*) to assess significant multiple-level interactions. Regression modeling was used to determine the directionality, magnitude and pattern of the change in participation of collegiate and junior men and women. For all analyses, a significance level of *P* < 0.05 was used to identify statistical significance.

## Results

### Collegiate rowing championships

#### Performance across years

**Fig 1:**
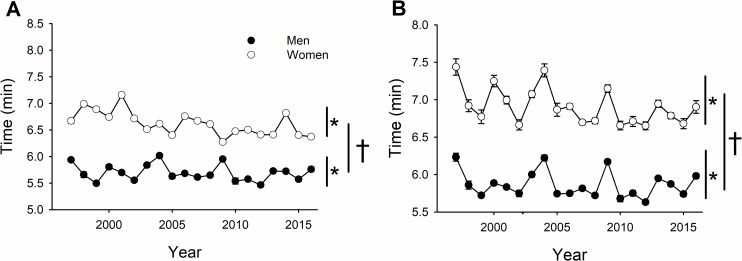
NCAA and IRA performance times of men and women improved across competition year [* denotes a significant effect of time, *p* < 0.001]; however, women improved more than men [† denotes a significant time × sex interaction, *P* = 0.001] for the heavyweight (A) and lightweight class (B). Note that heavyweight women compete in the NCAA and lightweight men and women and heavyweight men in the IRA.

Men had faster race times than women across all years, weight classes, and finishing places (Fig 1; 5.8 ± 0.2 vs 6.8 ± 0.4 min; sex effect, *P* < 0.001), and both sexes had faster race times across years of competition (Fig 1; year effect, *P* < 0.001). However, the women (both weight classes) improved more across years of competition from 1997 to 2016 than men (5.9% vs 3.5% improvement, respectively; year × sex, *P* = 0.001). As expected, heavyweight rowers had faster performance times than lightweight rowers (6.2 ± 0.5 vs. 6.4 ± 0.6 min; weight class effect, *P* < 0.001). Both heavyweight (Fig 1A) and lightweight (Fig 1B) rowers demonstrated an improvement in performance across time (year effect, *P* = 0.019) and this improvement was not different for heavyweights and lightweights (year × weight class, *P* = 0.906).

#### Performance across finishing place

**Fig 2:**
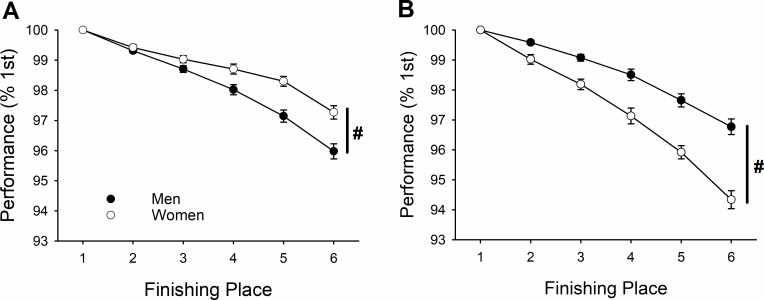
NCAA and IRA relative performance (% 1^st^ place) across finishing place for heavyweight (A) and lightweight class (B). The reduction in relative performance was greater for men than women [# denotes greater reduction in men, place × sex, *P* < 0.001] for the heavyweight class (A). Conversely, the reduction in relative performance was greater for women compared with men [† denotes greater reduction in women, place × sex, *P* < 0.001] for the lightweight class (B). Note that heavyweight women compete in the NCAA and lightweight men and women and heavyweight men in the IRA.

Finishing time (% 1^st^ place) declined across place for both men and women (Fig 2; 4.2 ± 3.2% reduction by 6^th^ place; place effect, *P* < 0.001), this reduction in performance was significant at every place after first (*pairwise comparison*, *P* < 0.005). This reduction in performance across place occurred for both weight classes (place effect, *P* < 0.001); however, the reduction in performance was greater for the lightweight class compared with heavyweight class (5.1 ± 4.3% vs 3.4 ± 1.2% reduction for 6^th^ place, respectively; place × weight class, *P* = 0.003).

Given the significant interaction of weight class, two post-hoc univariate ANOVAs (one for each weight class) with Bonferroni-corrected *p* value and sex as a between-subject factor were performed to examine the differences between men and women in the reduction of performance across finishing place. For the heavyweight class (Fig 2A), women had a *lesser* drop off in performance across finishing place compared with men (2.7 ± 1.0% vs 4.0 ± 1.1% reduction, respectively; place × sex, *P* < 0.001). For the lightweight class (Fig 2B), however, women had a greater relative drop off in performance compared with men (6.8 ± 5.4% vs 3.2 ± 1.2% reduction, respectively; place × sex, *P* < 0.001).

### Junior World Indoor Rowing Championships

#### Performance across years

**Fig 3:**
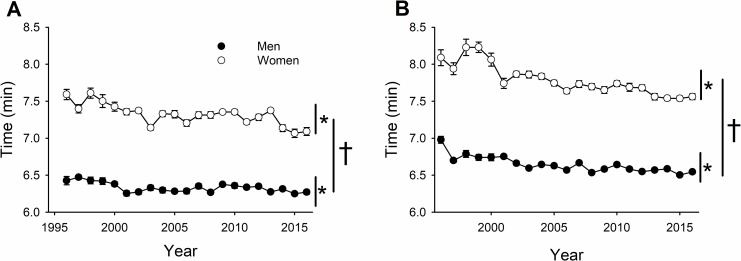
Ergometer performance times (“erg times”) of men and women in the Junior World Indoor Rowing Championships improved across competition year [* denotes significant time effect, *P* < 0.001]; although, women improved more than men [† denotes a significant time × sex interaction, *P* = 0.001] for the heavyweight (A) and lightweight class (B) during the World Indoor Championships.

Men had faster race times than women across all years, weight classes, and finishing places (Fig 3; 6.5 ± 0.2 vs. 7.5 ± 0.4 min, respectively; sex effect, *P* < 0.001), and both sexes improved in performance across the years (Fig 3; year effect, *P* < 0.001). However, the women improved more in performance than men with time between 1997 and 2016 (6.6% vs. 4.4% improvement, respectively; year × sex, *P* = 0.001). As expected, heavyweight rowers had faster performance times than lightweight rowers for all years combined (6.6 ± 0.6 vs. 7.0 ± 0.6 min, respectively; weight class effect, *P* < 0.001). Both heavyweight (Fig 3A) and lightweight (Fig 3B) rowers demonstrated a similar improvement in performance across time (year × weight class, *P* = 1.0).

#### Performance across finishing place

**Fig 4:**
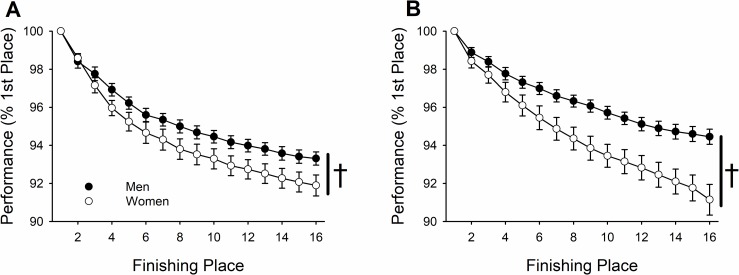
In the Junior World Indoor Rowing Championships, relative ergometer performance (% 1^st^ place) reduced across finishing place (place effect, *P* < 0.001); further, women had greater reductions compared to men [† denotes a significant place × sex interaction, *P* = 0.001] for heavyweight (A) and lightweight class (B).

When the finishing time of each place was considered relative to first place within each sex (% 1^st^ place), performance declined across place for both men and women (Fig 4; 6.5 ± 2.4% reduction by 16^th^ place; place effect, *P* < 0.001) and this reduction in performance was significant at every event place after first (*pairwise comparison*, *P* < 0.001). However, this reduction in performance across finishing place was greater for women compared with men (place × sex, *P* < 0.001). By 16^th^ place the men were 93.3 ± 1.6% of first place and the women 91.9 ± 2.5% in the heavyweights (Fig 4A). For the lightweights, by 16^th^ place, the men were 94.5 ± 1.8% of first place and the women 91.1 ± 3.7% (Fig 4B). The reduction in performance across finishing place was similar for the heavyweight class compared to the lightweight class (92.8 ± 2.0% vs. 93.1 ± 3.0% of first place, respectively, place × weight class, *P* = 0.993).

#### Sex difference across finishing place

**Fig 5:**
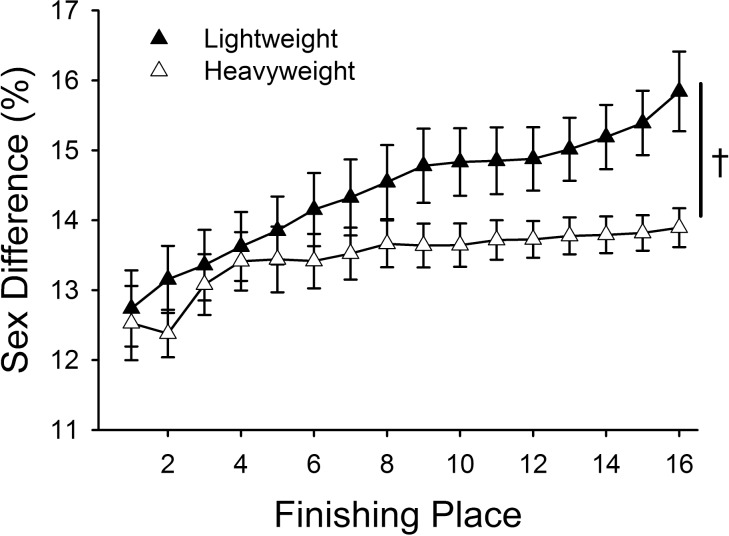
Sex difference in performance increased across finishing place for Junior World Indoor Championships [* denotes significant place effect, *P* < 0.001]; although, the sex difference was less for the heavyweight (open triangle) compared with the lightweight rowers (filled triangle) [† denotes a significant place × weight class interaction, *P* = 0.001] for top 16 place finishers.

Men had faster rowing times than women across all finishing places and both weight classes, thus there was a positive sex difference. However, the sex difference widened for both the heavyweight and lightweight classes across finishing place (place effect, *P* < 0.001; place × weight class, *P* = 0.372). The widening of the sex difference indicates that women had a greater drop in performance across finishing place relative to men (Fig 3). Additionally, the lightweight class had greater sex differences in performance than the heavyweight class (Fig 5; 14.5 ± 1.9% vs 13.8 ± 1.4% sex difference, respectively; weight class effect, *P* < 0.001).

### Participation in rowing

#### Collegiate on-water rowing

**Fig 6:**
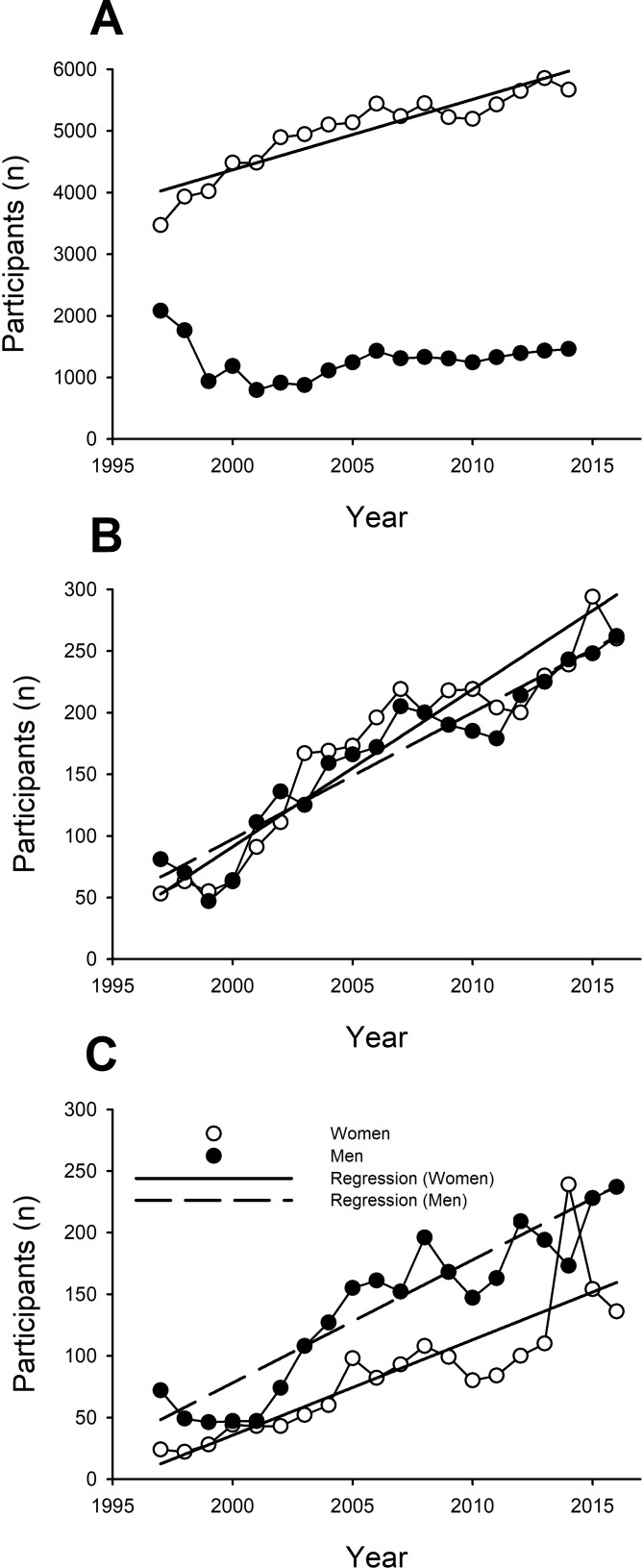
Participation of men (filled circles) and women (open circles) competing in the NCAA regardless of weight class (**A**), in the heavyweight division of Junior Championships (**B**), and in the lightweight division of the Junior Championships (**C**). **A:** Participation among the heavyweight women in the NCAA increased linearly (y = 113.1x + b, *r*^2^ = 0.847, *P* < 0.001); however, there was no significant regression among men in the NCAA. **B:** Participation increased among heavyweight men and women competing in the Junior Championships (y = 10.3x + b, *r*^2^ = 0.908, *P* < 0.001; y = 11.7x + b, *r*^2^ = 0.889, *P* < 0.001, respectively). **C:** Participation increased among lightweight men and women competing in the Junior Championships (y = 9.9x + b, *r*^2^ = 0.861, *P* < 0.001; y = 7.4x + b, *r*^2^ = 0.714, *P* < 0.001, respectively). (y = total number of participants, x = year of competition, b = constant).

Participation in collegiate rowing has steadily increased over time (Fig 6A). However, the increase in total participants is driven by the women. Participation among women has increased linearly (*r*^2^ = 0.847, *P* < 0.001) by ~113 women per year, whereas there has been no significant change (*P* = 0.899) in participation across years for collegiate men (Fig 6A).

#### Junior indoor rowing

Participation for juniors at the World Indoor Rowing Championships has steadily increased over time (Fig 6B and 6C). This increase in participation occurred for both men and women in heavyweight and lightweight classes. The participation among heavyweight women increased linearly (*r*^2^ = 0.889, *P* < 0.001) by ~12 women per year and for heavyweight men participation increased linearly (*r*^2^ = 0.908, *P* < 0.001) by ~10 men per year (Fig 6B). Similarly, the participation among lightweight women increased linearly (*r*^2^ = 0.714, *P* < 0.001) by ~8 women per year and participation for lightweight men participation increased linearly (*r*^2^ = 0.861, *P* < 0.001) by 10 men per year (Fig 6C).

## Discussion

The present results demonstrate for the first time in elite athletics that performance across place not only narrowed between men and women, but that women had faster times relative to first place at higher finishing places than men (Fig 2A). In addition, women improved more in performance across time than men between 1997 and 2016 for both the Collegiate Rowing (Fig 1) and Junior World Indoor Rowing Championships (Fig 3). Interestingly, in a recent review it was reported that no experimental manipulation or systematic historical comparison has convincingly shown a decrease in the sex difference between males and females [20]. In the current study, we show not only a decrease in the sex difference across years in collegiate and junior rowing, but that when performance is expressed as a percent of first place, women outperform men at higher finishing places in heavyweight collegiate rowing. Furthermore, we showed that the reduction in the sex difference in rowing performance was paralleled by an increase in participation of the women relative to the men in collegiate rowing. Overall, the findings suggest that decreased opportunities and participation are major contributors explaining a portion of the observed sex differences in elite athletic performance [9,10].

### Collegiate Rowing Championships

Men were faster than women among the top 6 finishers across all years (1997–2016) and both weight classes (heavy and light); thus, there was a significant sex difference in performance across all finishing places, years and weight classes (Fig 1). However, women improved more than men across the years of competition (Fig 1). Additionally, although performance of both men and women was reduced across finishing place as expected, this drop-off in performance across finishing place was *greater* for men compared with women for the heavyweight class (Fig 2A) but smaller for men compared with women for the lightweight class (Fig 2B). Accordingly, the collegiate women rowers increased in participation at ~113 per year over the time span that men showed no increases in the number of rowers (Fig 6). These findings are supportive of our primary hypothesis. Specifically, we hypothesized that given the increased opportunities and participation of women in rowing over the last two decades (Fig 6A) that we would find that the sex difference not only decreased but that performance of elite women rowers would show less of a drop-off than that for men from 1^st^ to 6^th^ place. This is the first report of men demonstrating a greater drop off in performance across finishing place compared to women; previous reports from swimming, triathlon, and short-, long- and ultra-distance running all demonstrate that women have a greater drop off in performance than men [8–15]. The only other report of a similar decrease in performance between men and women with finishing place was in the backstroke in swimming [8], which interestingly has also been reported to have greater participation rates by women than men [19]. Furthermore, because the benefits of rowing are disproportionately awarded to women in heavyweight rowing (e.g., athletic scholarships), we expected that the largest declines in the sex difference in performance would be for the heavyweights and not the lightweights, as was observed (Fig 2). The greater opportunity and financial benefit for heavyweight (and not lightweight) collegiate women relative to men are likely to underlie the better relative performance of women across finishing place. Interestingly, these differences occurred for the team sport of rowing, even though team sports require greater motivation to engage in cooperative group challenges, which is reported to be greater in males than females [21–23].

### Junior World Indoor Rowing Championships

Similar to the on-water performances during the Collegiate Rowing Championships, junior men were faster than women among the top 16 finishers in the ergometer performances across all years (1997–2016) and both weight classes (heavy and light; Fig 3A and 3B, respectively). Consistent with our secondary hypothesis, women improved more than men across the years of competition (Fig 3). However, contrary to this hypothesis, the greater improvement by the women across competition year was not different between the weight classes (Fig 3), and women had greater reductions in performance across finishing place compared to men for both weight classes (Fig 4). Both weight classes, however, had comparable increases in participation per year (Fig 6B and 6C) for the men and women (n = 8–12 per year). One potential rationale for different observations between the Collegiate Rowing Championships and the Junior World Indoor Rowing Championships is that the increased financial incentives for women (i.e. athletic scholarships) relative to men do not occur until these individuals are collegiate-level athletes. In addition, physical training and coaching expertise are also likely increased at the collegiate level and may additionally explain these sex-related differences between collegiate and junior rowers. Moreover, the growth in participation for both men’s and women’s rowing at the high school level was very similar (Fig 6B and 6C).

### Factors influencing the sex differences in sport performance

The potential role of psychosocial factors to influence sex differences in sport performance has been closely examined since the enactment and enforcement of Title IX in the United States [20]. It is reported that sex differences observed in sport performance are possibly due more to the evolved predisposition of men to be more competitive than women [16–18], which drives a reported greater interest and performance in sport among men compared to women [20]. A few different lines of evidence support this hypothesis, using surrogate assessments of ‘competitiveness’. First, although some previous studies report a decrease in the sex difference across time [12,25], others report a widening of the sex difference [15]. Second, in sports that involve running, the activity most commonly studied [17], the sex difference in participation has generally decreased up to the present time though there is still a large sex difference in the proportion of runners who show a primarily competitive rather than recreational orientation to running [16–18,26]. Third, the sex difference in performance widens with finishing place across nearly all sports that have been investigated including marathon and ultramarathon running, triathlon, and freestyle swimming [8–15], with the backstroke in swimming being the only exception with similar drop-offs for men and women with higher finishing place [8]. In addition, previous work [9,12] examining the relationships between increased participation of women in sport and reduced sex differences in performance have been largely correlational and therefore have not directly assessed causation.

The widening of the sex difference with finishing place has been used as support for the suggestion that men are innately more competitive than women [16]. Thus, given the widening sex difference with women outperforming men in the current study (Fig 2A), it may be tempting to consider if it is appropriate to suggest that women are more competitive than men. However, the better performance of women compared with men in terms of rowing is true only for heavyweight, collegiate women, but not lightweight, collegiate women nor junior women. Thus, it seems more probable that differences in opportunity and incentive systems can substantially impact observed sex differences in elite athletic performance. The increase in the number of collegiate women participants that we documented supports this idea. Regardless of the underlying psychosocial factors, there are innate physiological factors (e.g. stronger and faster muscle) that predispose men to better athletic performance than women [27], but within these differences participation and incentives appear to significantly influence sex differences.

The paucity of empirical support related to the decreased sex differences with increased participation by women, has been used to dismiss the underlying assumptions of Title IX [20]. Specifically, it is suggested that scholars are propagating incorrect views and that these views are influencing social policies [20]. For example, it is suggested that an underlying assumption of Title IX is that male and female sports interest is equal, or soon will be, and if this assumption is incorrect it could lead to suboptimal allocation of resources and possibly discrimination against males. Interestingly, the large growth of women’s, but not men’s, rowing over the last 20 years has been fueled by many different factors, not least of which is the need for many large schools that have American football programs to find a way to equalize participation between men’s and women’s sports at their institutions, a requirement of Title IX. Considering that it has taken 45 years since the passage of Title IX to find a significant reduction in the sex difference in sport performance, an equally valid question is to ask if society is doing enough to lead to a fair playing ground between men and women in terms of elite sport performance.

### Strengths and limitations

Data records for men’s and women’s rowing are easily available over the preceding 20-year span of time, though records are not complete preceding that timeframe. Moreover, although Title IX was passed in 1972, wide-scale enforcement of Title IX wasn’t implemented until the 90’s and many of the changes we observe today, for example in women’s rowing, are a product of just the changes enacted in the last 20 years. Although there are important socio-demographic characteristics that influence performance (e.g. age, training hours, and academic progress of student-athletes), the regulations enforced by collegiate rowing for men and women have been similar for the past 20 years and have resulted in relatively homogeneous populations and similar exposure to training both across this 20-year timeframe and between the sexes. Originally, we had planned to include men’s and women’s open (elite) and collegiate indoor rowing in our analyses from the World Indoor Rowing Championships, though it was clear from participation and performance data on these groups that elite performers were less likely to be competing in the World Indoor Rowing Championships more recently. Specifically, participation levels for elite women actually decreased around 2008 and average performance of the top 6 Junior women at the World Indoor Rowing Championships has actually exceeded that of elite women in 2014, likely indicating that collegiate and national team coaches are now less likely to send their best athletes to this indoor competition. Nonetheless, the strong showing by junior women, and the reliance of “erg time” as a recruiting tool by most colleges, justify this metric at the high school level, though future work could include data from satellite indoor events spread throughout the United States and overseas, as well as on-water data from junior competitions.

## Conclusions

In the current study, we show that collegiate heavyweight women had a lessor drop off than men in rowing time between the 1^st^ and 6^th^ placed boats. Specifically, when performance is expressed as a percent of first place, women outperform men at higher finishing places in heavyweight collegiate rowing: this is the first report of women having faster times than men across finishing place in any sport. We also show a steady decrease in the sex difference across years in collegiate rowing, which was paralleled by an increase in participation of the women relative to the men in collegiate rowing. Overall, our work suggests that decreased opportunities and participation are major contributors explaining at least part of the observed sex differences in elite athletic performance. Interestingly, these findings come from the sport of rowing, which historically is a male-dominated sport, with men’s rowing the first collegiate competition in the United States of America (i.e., Harvard-Yale Regatta in 1852). The fact that Title IX incentivized women’s rowing, and not men’s rowing, especially since the mid-1990’s when Title IX enforcement increased, has provided a unique testbed to evaluate sociological factors that influence sport performance.

## Abbreviations

ANOVA: Analysis of Variance
min: minutes
NCAA: National Collegiate Athletic Association
s: second
